# Long Non-coding RNA TUG1 Modulates Expression of Elastin to Relieve Bronchopulmonary Dysplasia via Sponging miR-29a-3p

**DOI:** 10.3389/fped.2020.573099

**Published:** 2020-10-30

**Authors:** Qinghua Zhong, Li Wang, Zhiye Qi, Jia Cao, Kun Liang, Caiying Zhang, Jiang Duan

**Affiliations:** ^1^Department of Pediatrics, First Affiliated Hospital of Kunming Medical University, Kunming, China; ^2^Department of Emergency, First Affiliated Hospital of Kunming Medical University, Kunming, China

**Keywords:** bronchopulmonary dysplasia, microRNAs, long non-coding RNA taurine up-regulated gene 1, miR-29a-3p, elastin

## Abstract

**Objective:** Multiple studies have highlighted that long non-coding RNAs (lncRNAs) may exert paramount roles in relieving bronchopulmonary dysplasia (BPD). The aim of our investigation is to probe the role and mechanism of lncRNA taurine upregulated gene 1 (TUG1) in BPD.

**Methods:** The current mouse model of BPD was simulated by induction of hyperoxia, and hyperoxia-induced mouse type II alveolar epithelial (MLE-12) (MLE-12) cells were established as a cellular model. Quantitative real-time polymerase chain reaction (qRT-PCR) was applied to determine relative expressions of TUG1, miR-29a-3p, and elastin (ELN). We assessed cell apoptosis by TdT-mediated dUTP-biotin nick-end labeling (TUNEL) staining. Western blot was used for detection of apoptosis-related proteins. Moreover, cell viability was tested by cell counting kit-8 (CCK-8) assay. Inflammatory factors were measured by enzyme-linked immunosorbent assay (ELISA). Dual-luciferase reporter (DLR) assay was employed to confirm relationship between genes.

**Results:** Upregulation of miR-29a-3p was found in lung tissues of BPD mice compared with lung tissues without BPD, while downregulations of TUG1 and ELN were discovered in BPD tissues in comparison with tissues without BPD. Increasing TUG1 was shown to alleviate lung injury of BPD mice and promote proliferation of hyperoxia-induced MLE-12 cells. Meanwhile, TUG1 inhibited inflammatory response and cell apoptosis in lung tissues of BPD mice and hyperoxia-induced MLE-12 cells. miR-29a-3p was targeted by TUG1 and negatively modulated by TUG1. ELN was inversely regulated by miR-29a-3p. Meantime, suppressive effects of TUG1 on apoptosis and inflammation were reversed by decreasing ELN or increasing miR-29a-3p in hyperoxia-induced MLE-12 cells.

**Conclusion:** lncRNA TUG1 relieved BPD through regulating the miR-29a-3p/ELN axis, which provided a therapeutic option to prevent or ameliorate BPD.

## Introduction

Bronchopulmonary dysplasia (BPD), a chronic lung disease of prematurity, is the most common respiratory disorder among infants born extremely preterm. The pathogenesis of BPD is multifactorial (1). Apart from postnatal determinants of lung injury and low gestational age, adverse antenatal factors, such as gestational hypertensive disorders, maternal obesity, and gestational diabetes, also lead to the pathological and clinical features of BPD (2–4). Currently, there are many studies to support several therapeutic strategies, including antenatal glucocorticoids, surfactant therapy, interleukin-1 receptor antagonist, and microbiome (5, 6). Despite intensifying efforts to manage BPD, BPD incidence and mortality of preterm neonates still pose a great challenge for us (7). Thus, raising novel prospects of therapy is essential for contributing to the development of BPD treatment.

Long non-coding RNAs (lncRNAs) comprising transcripts greater than 200 nucleotides are one of the emerging regulators, which lack the protein-coding function (8). In recent years, the roles of lncRNAs in BPD have attracted much attention (9). Cai et al. have reported that lncRNA MALAT1 can inhibit apoptosis of alveolar epithelial cells to protect preterm infants suffering from BPD (10). Mo et al. have stated that overexpression of lncRNA H19 promotes the progression of BPD by mediating MAPK pathway (11). Notably, lncRNA taurine upregulated gene 1 (TUG1) with 6.7 kb nucleotides is located at chromosome 22q12 (12). Growing literature has indicated that dysregulation of TUG1 is involved in lung-related diseases, such as chronic obstructive pulmonary disease (COPD) (13), pneumonia (14), and pulmonary arterial hypertension (PAH) (15). Nonetheless, there is no evidence clarifying the role of TUG1 in BPD.

MicroRNAs (miRNAs), endogenous non-coding RNAs with 18–23 nts in length, can participate in the posttranscriptional regulation of eukaryotic genes (16). Recently, miRNAs have been identified to implicate in the pathogenesis of BPD (17). Yuan et al. have revealed that inhibition of miR-421 can assist in the development of BPD (18). It has been found that miR-574-3p can protect the premature infants with BPD by regulating adrenomedullin (19). In a recent study, downregulation of miR-29a belonging to the miR-29 family (20) is reported to alleviate lung injury, promote cell proliferation, and repress cell apoptosis in BPD mice (21). In addition, miR-29b is demonstrated to be one component of a novel strategy to treat or prevent severe BPD (22). However, interaction between miR-29a-3p and TUG1 is not well-understood in BPD.

In the present study, we determined expression levels of TUG1, miR-29a-3p, and elastin (ELN). Subsequently, we delved into the role of TUG1 in BPD *in vivo* and *in vitro*. Mechanistically, interaction among TUG1, miR-29a-3p, and ELN was probed in hyperoxia-induced cells. Therefore, our study provided novel insight into the role of the TUG1/ miR-29a-3p/ELN axis in BPD and highlighted the potential of TUG1 to serve as a promising target for BPD therapy.

## Materials and Methods

### Animals

Newborn mice (C57BL/6 strain) without specific pathogen were purchased from the Experimental Animal Center of the Chinese Academy of Sciences (Shanghai, China). Mice were housed in a controlled environment with 12-h light–dark cycles and a humidity of 60% at 22–24°C. Mice were given free access to food and water. Approval of the study was obtained from the Animal Care and Use Committee of our hospital, and procedures of the animal experiment were complied with the National Institutes of Health Guide for the Care and Use of Laboratory Animals.

After the study, all mice were euthanized. Our right hand held the mouse tail and pulled it back, and our left thumb and forefinger pressed down firmly on the mouse head at the same time. The external force was used to dislocate the cervical spine of the mouse, and the spine and the brain were disconnected.

### Establishment of BPD Mice Models

Newborn mice within 12 h of birth were randomly selected regardless of gender and divided into the blank group (*n* = 12, weighing 3.03 ± 0.28 g), the hyperoxia group (*n* = 12, weighing 3.01 ± 0.42 g), the hyperoxia + negative control (NC) group (*n* = 12, weighing 3.01 ± 0.33 g), and the hyperoxia + TUG1 group (*n* = 12, weighing 3.02 ± 0.36 g). The adenovirus-packaged TUG1 vector and its corresponding NC were purchased from GenePharma (Shanghai, China). Before hyperoxia induction, mice were subcutaneously injected with adenovirus-packaged TUG1 vector of 5 μl or its control of 5 μl with reference to previous literature (21). Afterward, apart from the blank group, mice in other groups were subjected to hyperoxia treatment (continuous 5 l/min oxygen input with fraction of inspiration O_2_ > 90%) from the first day to the fourth day, then permitted to recover in room air for the following 10 days as previously reported (21). After hyperoxia treatment as described above for 14 days, mice were anesthetized with injection of phenobarbital (100 mg/kg) and euthanized. Serum samples were collected promptly after euthanization for enzyme-linked immunosorbent assay (ELISA), and lung tissues were resected for hematoxylin-eosin (HE) staining.

### HE Staining

The lung tissues from six mice of each group were fixed with 4% paraformaldehyde overnight at 4°C. Then the fixed tissues were embedded with paraffin and cut into serial sections. The sections were deparaffinized, hydrated, and stained with HE. Pathological changes of lung tissues were observed under a light microscope (Nikon, Tokyo, Japan). The extent of lung injury was assessed by lung injury scoring as previously presented (23). Five randomly selected fields of each slide were scored.

### TdT-Mediated dUTP-Biotin Nick–End Labeling Staining

The apoptosis in sections of lung tissues from six mice of each group was measured by using a TUNEL kit (Roche, Basel, Switzerland). In the first place, fractions of lung tissues and 50-μl solutions of TUNEL were mixed and reacted for 50 min. Afterward, the sections were incubated with 50 μl of peroxisome for 30 min at 37°C, and 100-μl working solutions of diaminobenzidine were used for color development. Next, the sections were counterstained by hematoxylin for 3 s and sealed by neutral resin. Tissue sections were observed using a fluorescence microscope (Olympus, Tokyo, Japan). The apoptotic cells were counted in five randomly selected fields from each group. The rate of positive cells was calculated by using the following formula: (the number of positive cells/the number of all cells) × 100%.

### Cell Culture and Transfection

A mouse type II alveolar epithelial (MLE-12) cell line was bought from the American Type Culture Collection (ATCC, Manassas, VA, USA). Cells were cultured in Dulbecco's modified Eagle's medium (DMEM; Gibco, USA) supplemented with 2% fetal bovine serum (FBS) in an incubator with 5% CO_2_ at 37°C. At 24 h after cell culture, MLE-12 cells were exposed to hyperoxia (95% O_2_ + 5% CO_2_) for 24 h (24), and MLE-12 cells exposed to normoxia (room air) acted as the control. When the cell density reached 70–80%, pcDNA-NC (RiboBio, Beijing, China), pcDNA-TUG1 (RiboBio), miR-NC (RiboBio), miR-29a-3p mimics (RiboBio), and small interfering (si)-ELN (RiboBio) were transfected for 48 h according to the instructions of Lipofectamine 3000 (Invitrogen, Carlsbad, CA, USA).

### Quantitative Real-Time Polymerase Chain Reaction

Total RNAs from lung tissues (12 mice of each group) and cells were isolated by using the Trizol kit (Thermo Fisher Scientific Inc., Waltham, MA, USA). The complementary DNA (cDNA) was synthesized with the Reverse Transcription kit (GenePharma), and the qRT-PCR experiment was performed by using the SYBR Green PCR kit (Takara, Dalian, China). The amplification program was listed as follows: 94°C for 10 min, 40 cycles of 95°C for 15 s, 60°C for 30 s, and 72°C for 45 s. All primers were bought from Takara. Primer sequences for qRT-PCR are shown in Table 1. Expressions of genes were standardized by using β-actin. The relative expressions of TUG1, miR-29a-3p, and ELN were calculated using the 2^ΔΔ^Ct method.

**Table 1:** Primers for quantitative real-time polymerase chain reaction (qRT-PCR).

**Gene**	**Forward**	**Reverse**
TUG1	5′-CTATACTCAGCTTCAGTGTT−3′	5′-TACTGTATGGCCACCACTCC.−3′
miR-29a-3p	5′-CGTAGCACCATCTGAAATCG−3′	5′- GTGCAGGGTCCGAGGT−3′
ELN	5′-GGCCATTCCTGGTGGAGTTCC−3′	5′-AACTGGCTTAAGAGGTTTGCCTCC−3′
β-actin	5′-ATCACTGCCACCCAGAAGAC−3′	5′-TTTCTAGACGGCAGGTCAGG−3′

### Western Blot

Total proteins were extracted from lung tissues (12 mice of each group) and cells using RIPA buffer (Beyotime, Shanghai, China). Proteins separated by 10% sodium dodecyl sulfate-polyacrylamide gels were transferred onto polyvinylidene fluoride (PVDF) membranes (Merck Millipore, Billerica, MA, USA), and the membranes were blocked with 5% skim milk for 1 h. After blocking, protein samples were incubated with primary antibodies anti-ELN (1:1,000, ab9519, Abcam, Cambridge, MA, USA), anti-caspase-3 (1:200, ab4051, Abcam), B-cell lymphoma 2 (Bcl-2; 1:1,000, ab196495, Abcam), and anti-β-actin (1:1,000, ab8227, Abcam) overnight at 4°C. After the membranes were washed with tris-buffered saline Tween (TBST), a secondary antibody (1:5,000, ab6728; Abcam) was added to incubate with the protein samples at 37°C for 2 h. An Alphalmager™2000 Imaging System (Alpha Innotech, San Leandro, USA) was applied to quantify the density of protein bands. The relative protein expressions of ELN, Bcl-2, and Caspase-3 were normalized by β-actin.

### ELISA

Transforming growth factor-β (TGF-β), interleukin 6 (IL-6), and IL-1β from cultured media or serum (12 mice of each group) were measured by using the corresponding ELISA kits (Abcam) as stated in the manufacturer's instructions. The optical density (OD) at 450 nm was measured using a Power Wave Microplate Reader (Bio-TEK, Vermont, USA).

### Cell Counting Kit-8

Cells were seeded in 96-well plates (5 × 10^3^ cells/well) and cultured for 24 h. Then CCK8 solutions (Beyotime) were added to each well, and cells were continuously nurtured at 37°C for 3 h. The optical density (OD) of each well at a wavelength of 450 nm was gauged by a microplate reader (Bio-Rad, Hercules, CA, USA).

### Dual-Luciferase Reporter Assay

The 3′-UTR fragment of TUG1 including the presumed binding sites of miR-29a-3p was inserted into pGL3-Basic (Promega, Shanghai, China) to construct the vector pGL3-TUG1-WT. The 3′-UTR fragment of TUG1 containing the mutant binding sites of miR-29a-3p was inserted into pGL3-Basic (Promega) to construct the vector pGL3-TUG1-MUT. The pGL3-reporter luciferase vectors of wild-type and mutant-type (Promega) holding the 3′-UTR sequence capable of binding miR-29a-3p of ELN were constructed. The above vectors were transfected into MLE-12 cells, together with miR-NC/mmu-miR-29a-3p (RiboBio) and miR-NC/miR-29a-3p mimics (RiboBio) for 48 h. DLR assay kit (Biovision, Milpitas, CA, USA) was used for detection of relative luciferase activity.

### Statistical Analysis

All data were analyzed using SPSS 22.0 software (IBM Corp., Armonk, NY, USA) and expressed as mean ± standard deviation. Comparisons among multiple groups were assessed by one-way ANOVA, followed by a Tukey's multiple comparison for pairwise comparisons. The *t*-test method was used for evaluating the differences between two groups. A *P* < 0.05 value was considered to be indicative of statistical significance. All experiments of the current study were repeated three times.

## Results

### Long Non-coding RNA Taurine Upregulated Gene 1 Was Downregulated in Bronchopulmonary Dysplasia Mice and It Alleviated Pulmonary Injury of Bronchopulmonary Dysplasia Mice

**Figure 1:**
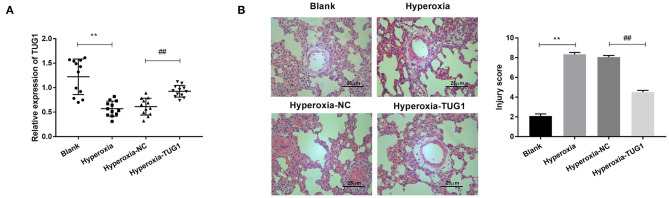
Long non-coding RNA (lncRNA) taurine upregulated gene 1 (TUG1) is downregulated in bronchopulmonary dysplasia (BPD) mice, and it alleviates pulmonary injury of BPD mice. **(A)** Relative expression of TUG1 was detected by quantitative real-time polymerase chain reaction (qRT-PCR) in lung tissues of BPD mice. **P < 0.01 vs. Blank. ^##^*P* < 0.01 vs. Hyperoxia-negative control (NC). **(B)** The injury score was used for assessing lung injury of mice. **P < 0.01 vs. Blank. ^##^*P* < 0.01 vs. Hyperoxia-NC. Each experiment was repeated three times.

We first induced BPD by exposure to hyperoxia. The result of qRT-PCR showed that TUG1 was markedly downregulated in the hyperoxia group relative to that in the blank group (*P* < 0.01) (Figure 1A), and relative expression of TUG1 in the hyperoxia-TUG1 group was evidently increased compared with the hyperoxia-NC group (*P* < 0.01) (Figure 1A). To clarify the function of TUG1 in BPD *in vivo*, pulmonary injury was assessed in BPD mice. As exhibited in Figure 1B, the injury score in the hyperoxia group was elevated in comparison with the blank group, and overexpression of TUG1 effectively inhibited pulmonary injury of BPD mice (*P* < 0.01) (Figure 1B).

### Overexpression of Taurine-Upregulated Gene 1 Inhibited Inflammation and Apoptosis in Lung Tissues of BPD Mice

**Figure 2:**
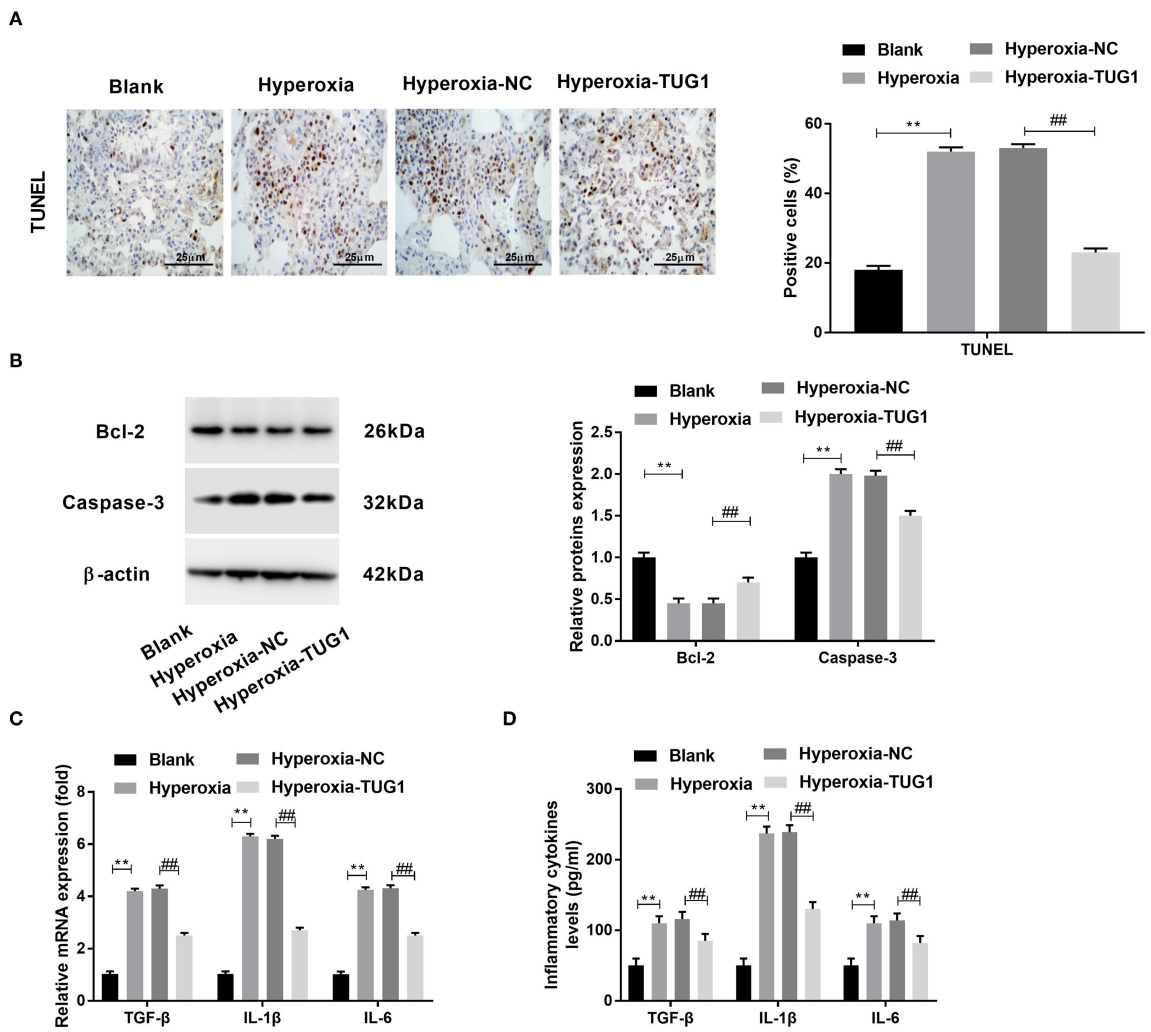
Overexpression of TUG1 inhibits apoptosis and inflammation in lung tissues of BPD mice. **(A)** TdT-mediated dUTP-biotin nick-end labeling (TUNEL) staining was used for detecting apoptotic cells. **P < 0.01 vs. Blank. ^##^*P* < 0.01 vs. Hyperoxia-negative control (NC). **(B)** Relative protein expressions of Caspase-3 and Bcl-2 in lung tissues of BPD mice were determined by Western blot. **P < 0.01 vs. Blank. ^##^*P* < 0.01 vs. Hyperoxia-NC. **(C)** Relative mRNA expressions of transforming growth factor-β (TGF-β), interleukin (IL)-6, and IL-1β in lung tissues of BPD mice were detected by quantitative real-time polymerase chain reaction (qRT-PCR). **P < 0.01 vs. Blank. ^##^*P* < 0.01 vs. Hyperoxia-NC. **(D)** TGF-β, IL-6, and IL-1β in the serum of BPD mice were measured by enzyme-linked immunosorbent assay (ELISA). **P < 0.01 vs. Blank. ^##^*P* < 0.01 vs. Hyperoxia-NC. Each experiment was repeated three times.

Then, we explored the effects of TUG1 on apoptosis and inflammation in BPD mice. The result of TUNEL staining demonstrated that apoptotic cells in lung tissues of BPD mice were increased compared with the blank group, and cell apoptosis of BPD mice was suppressed by TUG1 (*P* < 0.01) (Figure 2A). Besides, Western blot was performed to determine protein expressions of apoptosis-related proteins (Caspase-3 and Bcl-2). We observed that Caspase-3 was elevated by hyperoxia in lung tissues of mice, while Bcl-2 was decreased by hyperoxia in lung tissues of mice (*P* < 0.01) (Figure 2B). The upregulation of Caspase-3 and downregulation of Bcl-2 were reversed by overexpression of TUG1 in lung tissues of BPD mice (*P* < 0 01) (Figure 2B). To investigate the effect of TUG1 on inflammation of BPD mice, we measured levels of inflammatory cytokines. The results of ELISA and qRT-PCR displayed that TGF-β, IL-6, and IL-1β in the hyperoxia group were increased compared with the blank group, and TUG1 repressed the upregulations of TGF-β, IL-6, and IL-1β in the serum of BPD mice (all *P* < 0.01) (Figures 2C,D).

### Overexpression of TUG1 Could Suppress Apoptosis and Inflammatory Response, and Promote Proliferation of Hyperoxia-Induced Mouse Type II Alveolar Epithelial Cells

**Figure 3:**
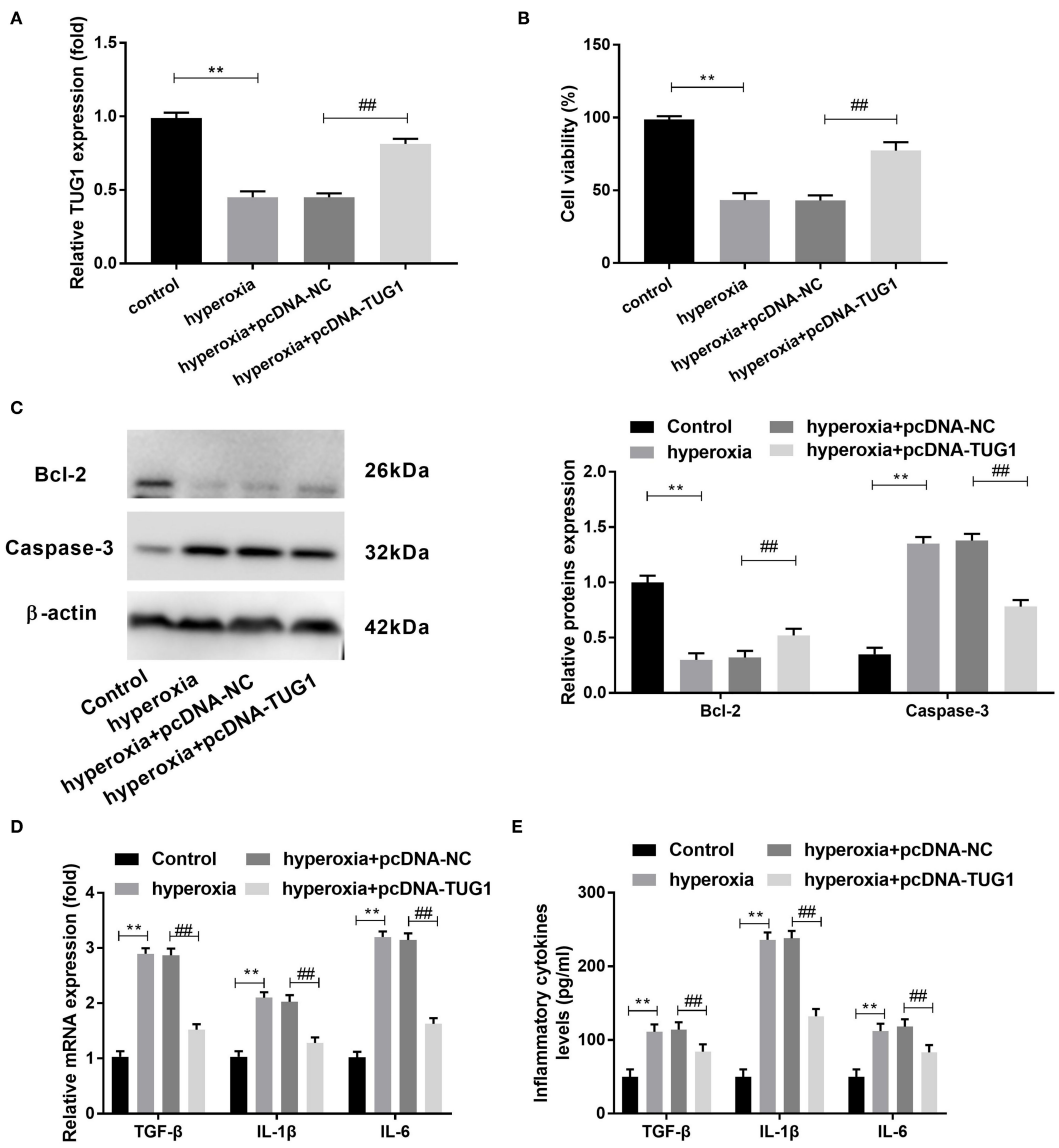
Increased TUG1 can suppress apoptosis and inflammatory response and promote proliferation of hyperoxia-induced MLE-12 cells. **(A)** Relative expression of TUG1 was detected by quantitative real-time polymerase chain reaction (qRT-PCR) in hyperoxia-induced MLE-12 cells. **P < 0.01, vs. control. ^##^*P* < 0.01 vs. hyperoxia + pcDNA-negative control (NC). **(B)** Cell viability was detected by cell counting kit-8 (CCK-8) assay in hyperoxia-induced MLE-12 cells. **P < 0.01 vs. control. ^##^*P* < 0.01 vs. pcDNA-NC. **(C)** Relative protein expressions of Caspase-3 and Bcl-2 were determined by Western blot in hyperoxia-induced MLE-12 cells. **P < 0.01, vs. Control. ^##^*P* < 0.01 vs. hyperoxia + pcDNA-NC. **(D)** Relative mRNA expressions of transforming growth factor-β (TGF-β), interleukin (IL)-6, and IL-1β in hyperoxia-induced MLE-12 cells were detected by qRT-PCR. **P < 0.01 vs. Control. ^##^*P* < 0.01 vs. hyperoxia + pcDNA-NC. **(E)** TGF-β, IL-6, and IL-1β in cultured media were measured by enzyme-linked immunosorbent assay (ELISA). **P < 0.01 vs. Control. ^##^*P* < 0.01 vs. hyperoxia + pcDNA-NC. Each experiment was repeated three times.

Subsequently, we probed the function of TUG1 in hyperoxia-induced MLE-12 cells. We found that there was a downregulation of TUG1 in hyperoxia-induced MLE-12 cells compared with MLE-12 cells without hyperoxia treatment, and transfection of pcDNA-TUG1 elevated the expression of TUG1 in hyperoxia-induced MLE-12 cells (all *P* < 0.01) (Figure 3A). The result of CCK-8 assay revealed that cell viability was repressed by hyperoxia in MLE-12 cells, and cell viability of MLE-12 cells treated with hyperoxia was promoted by overexpression of TUG1 (all *P* < 0.01) (Figure 3B). MLE-12 cells induced by hyperoxia showed the upregulation of Caspase-3 and downregulation of Bcl-2, and effects of hyperoxia on Caspase-3 and Bcl-2 were reversed by overexpression of TUG1 (all *P* < 0.01) (Figure 3C). Besides, our data indicated that TGF-β, IL-6, and IL-1β in cultured media were significantly augmented by hyperoxia at both mRNA and protein levels (all *P* < 0.01) (Figures 3D,E). Upregulation of TUG1 suppressed the production of TGF-β, IL-6, and IL-1β at both mRNA and protein levels in MLE-12 cells treated with hyperoxia (all *P* < 0.01) (Figures 3D,E).

### miR-29a-3p Was a Target Gene of TUG1 and Inversely Regulated by TUG1

**Figure 4:**
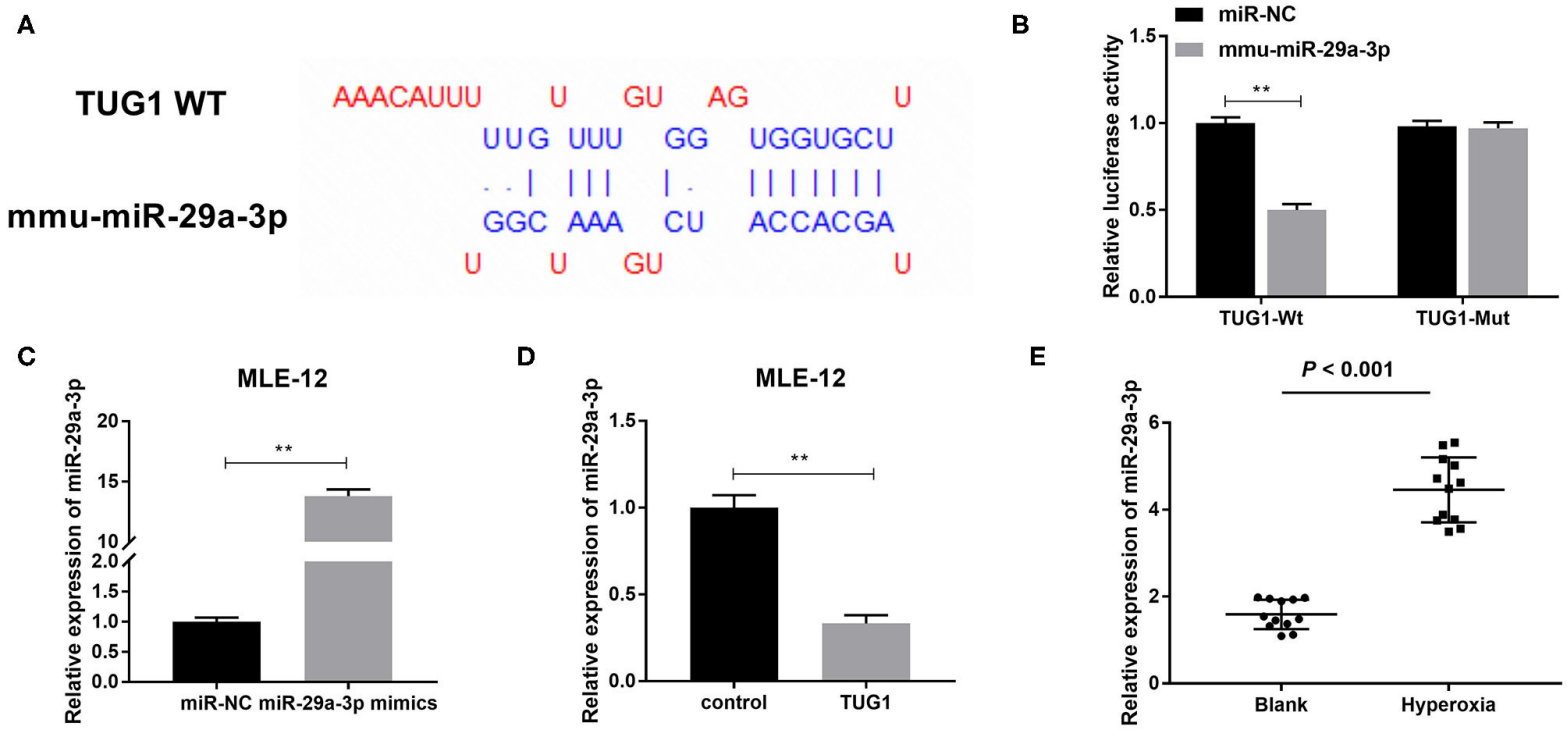
miR-29a-3p is a target gene of TUG1 and inversely regulated by TUG1. **(A)** The binding sites between TUG1 and miR-29a-3p were predicted by starbase2.0. **(B)** Relative luciferase activity of TUG1 vector was detected by dual-luciferase reporter (DLR) assay. **P < 0.01 vs. miR-negative control (NC). **(C)** After transfection of miR-29a-3p mimics, relative expression of miR-29a-3p was detected by quantitative real-time polymerase chain reaction (qRT-PCR) in hyperoxia-induced MLE-12 cells. **P < 0.01 vs. miR-NC. **(D)** After transfection of TUG1, relative expression of miR-29a-3p was detected by qRT-PCR in hyperoxia-induced MLE-12 cells. **P < 0.01 vs. miR-NC. **(E)** Relative expression of miR-29a-3p was detected by qRT-PCR in lung tissues of BPD mice. *P* < 0.001 vs. Blank. Each experiment was repeated three times.

In order to further delve to the mechanism of TUG1 in hyperoxia-induced MLE-12 cells, we predicted target genes of TUG1 by starbase2.0. miR-29a-3p was identified as a target of TUG1 with putative binding sites (Figure 4A). To test this hypothesis, a DLR assay was performed and demonstrated that relative luciferase activity of TUG1-Wt vector was remarkably lower in MLE-12 cells transfected with mmu-miR-29a-3p than that in MLE-12 cells transfected with miR-NC (*P* < 0.01) (Figure 4B). By contrast, relative luciferase activity had no significant difference after the introduction of mmu-miR-29a-3p in MLE-12 cells transfected with the TUG1-Mut vector (Figure 4B). Furthermore, we noted that miR-29a-3p mimics significantly elevated the relative expression of miR-29a-3p in hyperoxia-induced MLE-12 cells (*P* < 0.01) (Figure 4C). Overexpression of TUG1 markedly decreased the expression of miR-29a-3p in hyperoxia-induced MLE-12 cells (*P* < 0.01) (Figure 4D). In the current mouse model of BPD, we discovered that miR-29a-3p in the hyperoxia group was highly expressed in comparison with the blank group (*P* < 0.001) (Figure 4E).

### ELN Was a Downstream Target of miR-29a-3p and Negatively Regulated by miR-29a-3p

**Figure 5:**
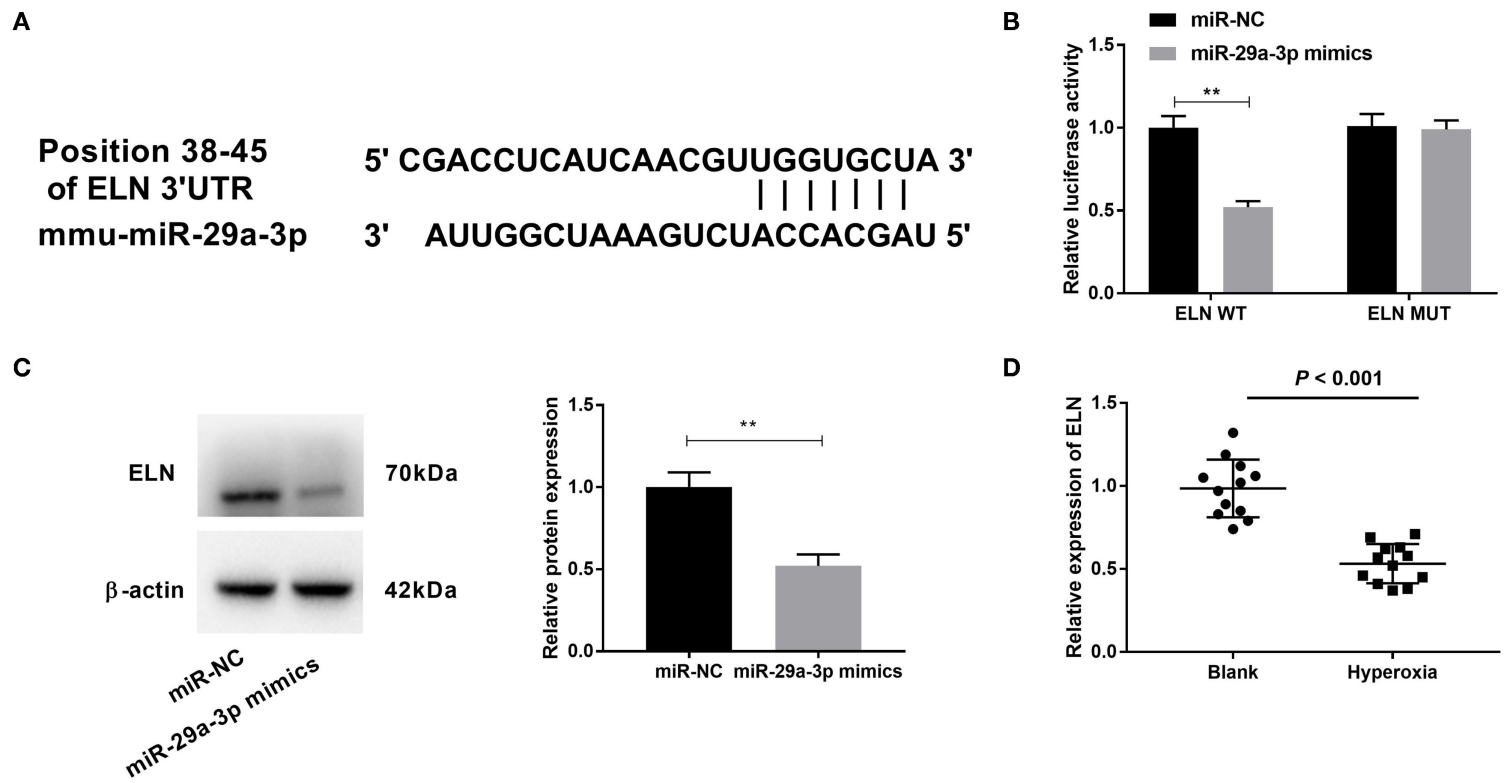
Elastin (ELN) is a downstream target of miR-29a-3p and negatively correlated with miR-29a-3p. **(A)** The binding sites between miR-29a-3p and ELN were predicted by starbase2.0. **(B)** Relative luciferase activity of ELN vector was detected by DLR assay. **P < 0.01 vs. miR-negative control (NC). **(C)** After transfection with miR-29a-3p mimics, expression of ELN was detected by Western blot in hyperoxia-induced MLE-12 cells. **P < 0.01 vs. miR-NC. **(D)** Quantitative real-time polymerase chain reaction (qRT-PCR) was used to detect relative expression of ELN in lung tissues of BPD mice. *P* < 0.001 vs. Blank. Each experiment was repeated three times.

To further examine the mechanism of TUG1/miR-29a-3p in hyperoxia-induced MLE-12 cells, we employed starbase2.0 to predict the downstream targets of miR-29a-3p, which showed that ELN contained the binding sequence for miR-29a-3p (Figure 5A). Then DLR assay confirmed the cooperation between miR-29a-3p and ELN. We found that miR-29a-3p mimics reduced the relative luciferase activity of the ELN WT reporter (*P* < 0.01) (Figure 5B), but miR-29a-3p mimics had no effect on ELN MUT reporter in MLE-12 cells (Figure 5B). Furthermore, relative protein expression of ELN was decreased by transfection of miR-29a-3p mimics in hyperoxia-induced MLE-12 cells (*P* < 0.01) (Figure 5C). In lung tissues of hyperoxia-induced mice, relative expression of ELN in the hyperoxia group was lower than that in the blank group (*P* < 0.001) (Figure 5D).

### lncRNA TUG1 Suppressed Apoptosis and Inflammation via Regulating miR-29a-3p/ELN Axis in Hyperoxia-Induced MLE-12 Cells

**Figure 6:**
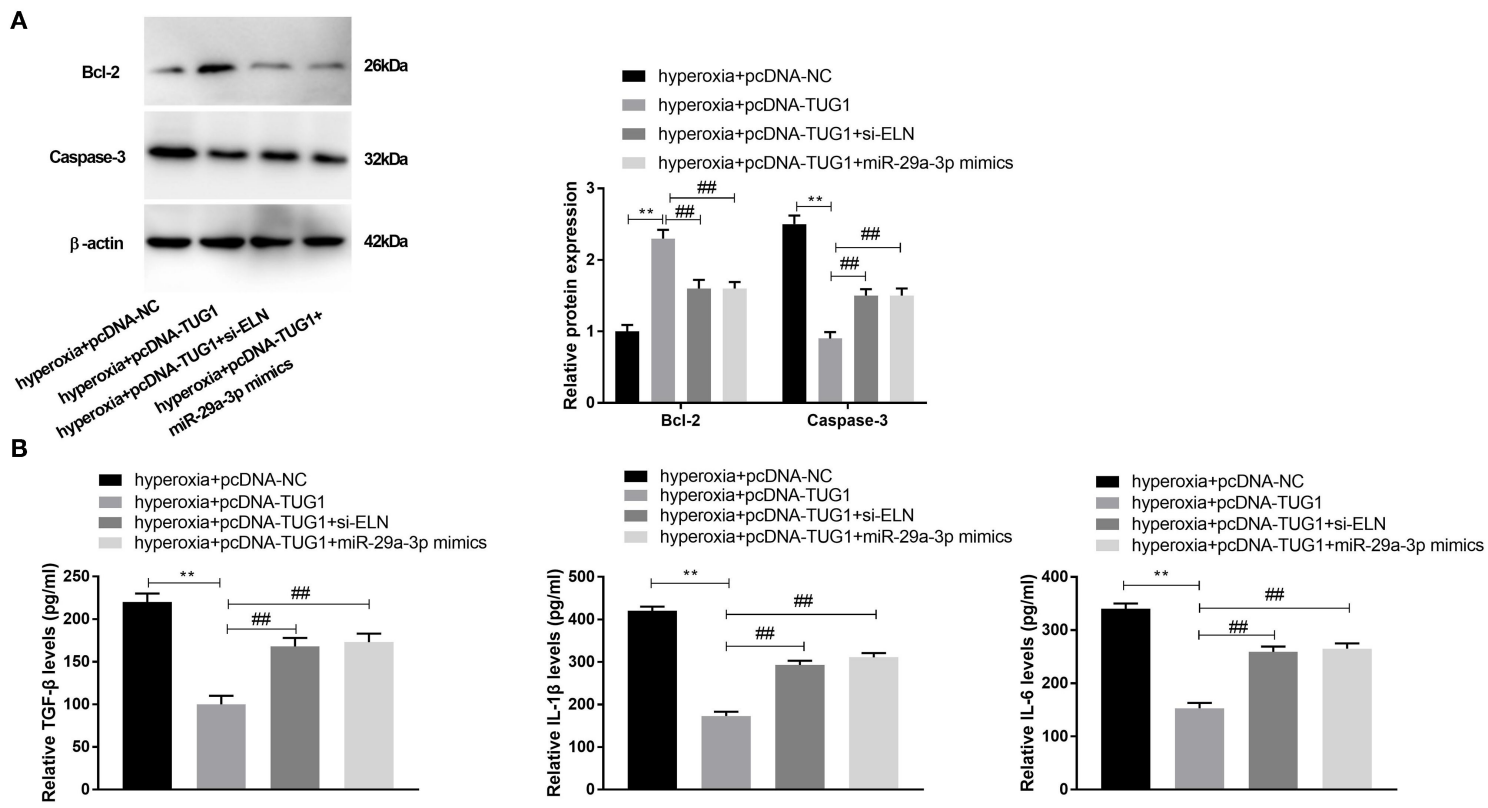
lncRNA TUG1 represses apoptosis and inflammation through regulating miR-29a-3p/ELN axis in hyperoxia-induced MLE-12 cells. **(A)** Relative protein expressions of Bcl-2 and Caspase-3 in hyperoxia-induced MLE-12 cells were detected by Western blot. **P < 0.01 vs. hyperoxia + pcDNA-negative control (NC). ^##^*P* < 0.01 vs. hyperoxia + pcDNA-TUG1. **(B)** Relative levels of TGF-β, IL-6, and IL-1β in cultured media were measured by ELISA. **P < 0.01 vs. hyperoxia + pcDNA-NC. ^##^*P* < 0.01 vs. hyperoxia + pcDNA-TUG1. Each experiment was repeated three times.

To verify the regulatory relationship among TUG1, miR-29a-3p, and ELN, rescue experiments were implemented. We discovered that transfection of pcDNA-TUG1 dramatically promoted the expression of Bcl-2, while suppressing the expression of Caspase-3 in hyperoxia-induced MLE-12 cells (*P* < 0.01) (Figure 6A). Meantime, the promotion effect of TUG1 on Bcl-2 and the suppression effect of TUG1 on Caspase-3 were reversed by downregulation of ELN or upregulation of miR-29a-3p hyperoxia-induced MLE-12 cells (*P* < 0.01) (Figure 6A). The result of ELISA showed that TUG1 inhibited the production of TGF-β, IL-6, and IL-1β, and the inhibition effects of TUG1 on TGF-β, IL-6, and IL-1β of MLE-12 cells were reversed by overexpression of miR-29a-3p or knockdown of ELN in MLE-12 cells induced by hyperoxia (all *P* < 0.01) (Figure 6B).

## Discussion

BPD leads to multiple organ dysfunctions and remains a chief threat to premature infants all over the world (25). Nowadays, there is compelling evidence highlighting the crucial roles of lncRNAs in the pathogenesis of BPD (10, 11). To explore the role of lncRNA TUG1 in BPD *in vivo*, the current mouse model of BPD was established by the induction of hyperoxia in our study referring to previous studies (26–28). In this model, mice were exposed to hyperoxia (>90% oxygen) for 4 days to repress the development of lung bronchium at the saccular stage and then exposed to normoxia for the next 10 days to recover the development of lung bronchium at the alveolar stage. This model in mice can well simulate human BPD to some extent. In this study, obvious pulmonary injury was found in BPD mice, indicating that the current mouse model of BPD was established successfully.

In recent years, lncRNA TUG1 emerges as a new player in gene regulation in lung-related diseases (29–31). Many studies have indicated that TUG1 is downregulated in lung-related diseases, including acute lung injury (ALI) (29) and non-small cell lung cancer (NSCLC) (32). Similar to the above studies, we also observed the downregulation of TUG1 in BPD tissues and hyperoxia-induced cells. Furthermore, the regulatory role of TUG1 on inflammation have been widely explored in COPD (33) and ALI (29). Qiu et al. have revealed that TUG1 shows a protective effect on LPS-induced primary murine pulmonary microvascular endothelial cells and ameliorates sepsis-induced inflammation and pulmonary injury in mice (29). Moreover, TUG1 is reported to alleviate LPS-evoked inflammatory response in pneumonia (14). Similar to the results of the above literature, we also found that overexpression of TUG1 alleviated inflammation and pulmonary injury of BPD mice. *In vitro*, TUG1 suppressed inflammation of hyperoxia-induced MLE-12 cells. Our results suggested that TUG1 could inhibit hyperoxia-induced inflammation *in vivo* and *in vitro*. In addition, Das et al. have stated that cell apoptosis exerts a critical role in the process of BPD (34). Analogously, we also probed apoptosis in BPD tissues and hyperoxia-induced cells. We found that that TUG1 suppressed cell apoptosis in lung tissues of BPD mice and hyperoxia-induced MLE-12 cells and promoted proliferation of hyperoxia-induced MLE-12 cells. In addition, our findings were supported by the following two studies. Overexpression of TUG1 suppresses cell apoptosis in mice with lung injury (29). TUG1 enhances the proliferative ability of human pulmonary smooth muscle cells (HPASMCs) in PAH (15), implying the antiapoptotic role for TUG1 in PAH. As mentioned above, we deduced that TUG1 extenuated BPD by inhibiting inflammation and apoptosis.

Considering that involvement of several miRNAs in the early development of the lungs is well-known, the imperative role of miR-29 family in BPD has attracted much attention recently (35). Prior literature has declared that miR-29 acts as a potential target for BPD treatment (22). Hu et al. have revealed that inhibition of miR-29a can alleviate lung injury, promote cell proliferation, and repress cell apoptosis in BPD mice model (21). Besides, it has been indicated that upregulation of miR-29a is evident in mice with BPD (21). In line with a previous study, we discovered that miR-29a-3p was highly expressed in BPD mice relative to that in mice without BPD, indicating that miR-29a-3p might participate in the development of BPD. However, a study from Durrani-Kolarik et al. has indicated that upregulation of miR-29b mitigates alveolarization of lung tissues in a neonatal mouse model of BPD induced by hyperoxia (22). Because miR-29a-3p and miR-29b are different isoforms of the miR-29 family, they may exert different functions in BPD. Increasing studies have demonstrated that TUG1 is involved in the progression of pulmonary injury and hypoxic pulmonary hypertension (HPH) via sponging miR-127 (14) and miR-374c (36). In the current study, we found that miR-29a-3p acted as a downstream target of TUG1 in hyperoxia-induced MLE-12 cells, which was inversely regulated with TUG1. Moreover, we found that overexpression of miR-29a-3p reversed the suppression effects of TUG1 on apoptosis and inflammation in hyperoxic cells. Taken together, our demonstration implied that TUG1 alleviated BPD through sponging miR-29a-3p *in vitro*.

ELN, an extracellular matrix (ECM) protein, is reported to allow the large arteries to reversibly expand and relax with every cardiac cycle (37). It has been reported that the expression of ELN is decreased in a primate model of BPD (38). Abnormal expression of ELN is associated with BPD (39). Similar to the above results, we also observed that ELN was notably decreased in lung tissues of hyperoxia-induced BPD mice. Based on the above outcomes, we inferred that ELN was relevant to BPD. Furthermore, previous studies have confirmed that ELN is targeted by miR-145 in the fibrosis of pulmonary fibroblasts (40) and by miR-29 in mediating offspring lung phenotype (41). In the present study, we discovered that ELN was targeted by miR-29a-3p, and it was negatively regulated by miR-29a-3p in the lung tissues of BPD mice. Given the above outcomes, we speculated that overexpression of miR-29a-3p might exert its role by targeting ELN in hyperoxic cells *in vitro*. Meanwhile, we found that the inhibitory effects of TUG1 on inflammation and apoptosis in hyperoxia-induced MLE-12 cells were reversed by knockdown of ELN. At length, we concluded that TUG1 might relieve BPD by mediating miR-29a-3p/ELN axis *in vitro*.

In summary, TUG1 was downregulated in lung tissues of BPD in comparison with lung tissues without BPD. Overexpression of TUG1 was shown to alleviate lung injury of BPD mice *in vivo*. miR-29a-3p was targeted by TUG1, and ELN was inversely regulated by miR-29a-3p. In general, our report uncovered that TUG1 repressed apoptosis and inflammation of hyperoxia-induced MLE-12 cells via mediating the miR-29a-3p/ELN axis. This newly identified axis provided a novel strategy for the prevention and treatment of BPD.

## Data Availability Statement

All datasets presented in this study are included in the article/supplementary material.

## Ethics Statement

The ethics committee of First Affiliated Hospital of Kunming Medical University.

## Author Contributions

QZ was responsible in the conception, design, and analysis of data, performed the data analyses, and wrote the manuscript. LW contributed to the conception of the study and wrote the manuscript. ZQ contributed significantly to the analysis, manuscript preparation, and wrote the manuscript. JC performed the data analyses and wrote the manuscript. KL wrote the manuscript. CZ performed the data analyses and wrote the manuscript. JD contributed significantly to analysis and manuscript preparation, and wrote the manuscript. QZ, LW, ZQ, JC, CZ, JD, and KL carried out the experiments. All authors have read and approved the manuscript.

## Conflict of Interest

The authors declare that the research was conducted in the absence of any commercial or financial relationships that could be construed as a potential conflict of interest.
